# Heat Shock Transcription Factors as Central Integrators of Plant Stress Responses: From Thermotolerance to Multi-Stress Resilience

**DOI:** 10.3390/biology14121800

**Published:** 2025-12-18

**Authors:** Yuan Li, Kang Gong, Xinyi Wang, Zhihong Sun, Fei Ding

**Affiliations:** College of Agriculture and Biology, Liaocheng University, Liaocheng 252000, China; 2310150212@stu.lcu.edu.cn (Y.L.); 2510190206@stu.lcu.edu.cn (K.G.); 2510190242@stu.lcu.edu.cn (X.W.)

**Keywords:** heat shock transcription factors (HSFs), abiotic stress, drought tolerance, cold stress, salinity, ROS homeostasis, transcriptional regulation, plant resilience

## Abstract

Heat shock transcription factors (HSFs) have long been recognized for their essential role in mediating thermotolerance via the activation of heat shock proteins (HSPs). Recent studies, however, have significantly broadened this view, revealing that HSFs function as versatile transcriptional regulators orchestrating plant adaptation to a wide range of abiotic and biotic stresses. This review synthesizes current knowledge of HSF structure, activation, and canonical roles in the heat shock response, while emphasizing emerging insights into their diverse functions beyond heat stress. Evidence from both model and crop species demonstrates that many HSFs confer tolerance to a broad range of stresses, including drought, cold, salinity, oxidative stress, and pathogen attack, through intricate crosstalk with hormonal (e.g., ABA, SA, JA) and redox signaling pathways, as well as MAPK-mediated phosphorylation. We also discuss biotechnological strategies such as CRISPR/Cas-mediated genome editing, stress-inducible promoter engineering, and synthetic transcriptional circuits that offer promising avenues for fine-tuning HSF expression and enhancing multi-stress resilience in crops. A deeper understanding of HSF multifunctionality not only advances our comprehension of plant stress biology but also provides a foundation for engineering resilient crops in the context of global climate change.

## Simple Summary

Plants are constantly faced with environmental challenges like extreme heat, drought, and diseases, which threaten their growth and our food supply. This review explores a special family of plant proteins, known as heat shock transcription factors, which act as master switches inside plant cells. While they were first known for helping plants survive heat by activating protective molecules, we now know their role is much broader. This article summarizes recent discoveries showing that these master switches are central to plant resilience against a wide variety of stresses, including drought, salinity, cold, and pathogen attacks. They achieve this by coordinating complex communication networks with the plant’s internal hormone and antioxidant systems. We conclude that understanding and harnessing the power of these proteins offers a promising biotechnology strategy to engineer crops that can better withstand the multiple challenges posed by a changing climate, ultimately contributing to future global food security.

## 1. Introduction

Plants are continuously exposed to a fluctuating environment that imposes multiple types of abiotic and biotic stresses, such as high temperature, drought, salinity, cold, and pathogen attack [1,2,3,4,5,6]. These stresses often occur simultaneously or sequentially, leading to complex physiological and molecular responses that ultimately determine plant growth, productivity, and survival. Understanding how plants sense, integrate, and respond to these diverse stresses is a central question in plant biology. This is particularly relevant in the context of global climate change, which is predicted to intensify temperature extremes and water deficits worldwide [7,8].

Heat shock transcription factors (HSFs) represent one of the most evolutionarily conserved and functionally versatile transcription factor families in plants. Initially discovered for their role in the heat shock response (HSR), HSFs activate the transcription of heat shock proteins (HSPs) that function as molecular chaperones to maintain protein homeostasis under elevated temperatures [9,10]. However, accumulating evidence reveals that HSFs are not confined to thermal stress; rather, they participate in a wide spectrum of physiological processes, including responses to drought, cold, salinity, oxidative stress, heavy metal toxicity, and pathogen attack, as well as developmental regulation and hormone signaling [11,12,13].

The diversification and expansion of the HSF gene family across plant species have enabled a sophisticated transcriptional network that orchestrates cellular defense, growth, and metabolic adaptation. For example, *Arabidopsis thaliana* contains 21 HSF genes classified into three major classes, including A, B, and C, each with distinct structural motifs and regulatory functions [14,15]. Crop species such as maize, soybean, and wheat harbor even larger HSF families, reflecting adaptive specialization in response to ecological and evolutionary pressures [16,17,18].

The functional plasticity of HSFs arises from several unique features: (i) modular domain architecture that allows DNA-binding, oligomerization, and regulatory control; (ii) multilayered regulation at transcriptional, post-transcriptional, and post-translational levels; and (iii) cross-talk with diverse signaling pathways, including reactive oxygen species (ROS), abscisic acid (ABA), salicylic acid (SA), ethylene, and brassinosteroids [15,19,20,21]. Furthermore, the hierarchical and cooperative action of multiple HSFs, where master regulators such as HSFA1 activate downstream HSFs and stress-responsive genes, illustrates a complex regulatory cascade that fine-tunes stress adaptation [22,23]. Recent advances in transcriptomics, proteomics, and systems biology have expanded our understanding of HSF-mediated networks, revealing their involvement in stress memory, epigenetic regulation, and developmental plasticity [24,25,26]. These findings highlight that HSFs function as central nodes integrating environmental and developmental signals to balance stress protection with growth optimization.

This review provides a comprehensive overview of the molecular architecture, regulatory mechanisms, and functional diversity of plant HSFs, emphasizing their roles beyond thermotolerance. We first summarize their structural organization and modes of regulation, followed by their canonical function in the heat stress response. We then discuss their emerging functions in other abiotic and biotic stresses. We also explore their potential applications in crop improvement. Emerging research on HSF-mediated stress memory, epigenetic regulation, and synthetic biology approaches opens new frontiers for engineering climate-resilient crops. Finally, we conclude with perspectives on future research directions and the broader implications of HSFs as central integrators of plant resilience. Deciphering how HSFs integrate multi-stress signals provides not only mechanistic insights but also a roadmap for engineering sustainable crop resilience.

## 2. Molecular Architecture and Regulation of Plant HSFs

### 2.1. Structural Organization, Classification and Evolutionary Diversification

#### 2.1.1. Structural Domains

**Figure 1:**
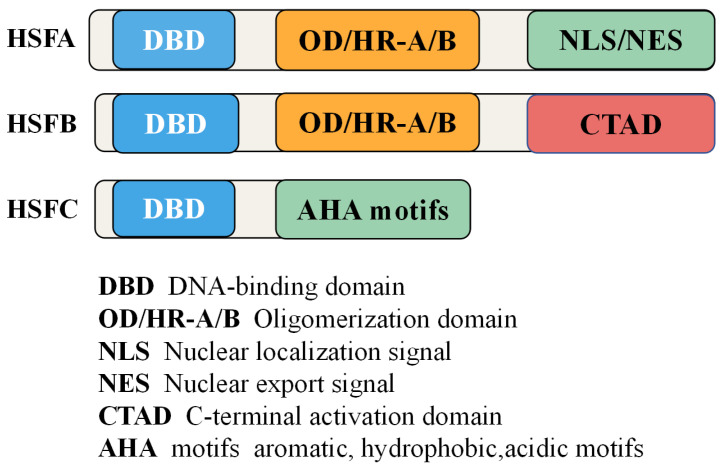
Structural organization of HSFs. HSFs share a conserved modular architecture comprising (1) an N-terminal DNA-binding domain (DBD) for recognition of heat shock elements (HSEs), (2) an oligomerization domain (HR-A/B) responsible for trimer formation, (3) nuclear localization (NLS) and export signals (NES) for nucleocytoplasmic shuttling, and (4) a C-terminal activation domain (CTAD) containing aromatic, hydrophobic, and acidic (AHA) motifs in HSFA-type HSFs. HSFBs typically lack AHA motifs and act as co-regulators, while HSFCs display intermediate features. The domain arrangement underlies functional specialization and regulatory diversity across plant species.

Heat shock transcription factors (HSFs) are defined by a highly conserved modular structure that underpins their function as transcriptional regulators. The core structural organization includes five principal domains: the DNA-binding domain (DBD), the oligomerization domain (OD) or heptad repeat region (HR-A/B), the nuclear localization signal (NLS), the nuclear export signal (NES), and the C-terminal activation domain (CTAD) (Figure 1) [13,15].

The DBD, typically located at the N-terminus, is characterized by a helix-turn-helix motif and a β-sheet wing, enabling specific recognition of heat shock elements (HSEs) in target promoters. HSEs consist of inverted repeats of the 5′-nGAAn-3′ motif, often organized as contiguous or gapped trimeric arrays [27]. This domain shows remarkable conservation across eukaryotes, underscoring its fundamental role in DNA binding and promoter specificity.

Adjacent to the DBD lies the OD (HR-A/B), which mediates trimerization, a prerequisite for HSF activation. The OD contains hydrophobic heptad repeats that form a coiled-coil structure, enabling both homo- and hetero-oligomerization among HSF family members [15,28]. The central HR-A/B domain is separated by variable-length insertions that define the three major classes of plant HSFs (A, B, and C). HSFA proteins typically contain longer linkers between HR-A and HR-B, while HSFBs possess shorter ones, influencing their regulatory behavior and interaction dynamics [14].

#### 2.1.2. Functional Distinctions of HSF Classes

The C-terminal region harbors regulatory motifs essential for transactivation and signal integration. In HSFA members, this region typically contains AHA motifs (aromatic, hydrophobic, acidic amino acids), which function as activation domains by recruiting transcriptional machinery and co-activators [29]. In contrast, HSFB members generally lack AHA motifs and may act as co-repressors, modulating transcriptional activity through protein–protein interactions or competition for HSE binding [30]. Class C HSFs, less well-characterized, often exhibit hybrid or intermediate properties, potentially participating in specialized stress responses or developmental regulation [31,32].

#### 2.1.3. Evolutionary Diversification

Phylogenetic analyses across plant lineages, ranging from algae to angiosperms, indicate that the HSF gene family has undergone extensive duplication, subfunctionalization, and neofunctionalization during evolution [33,34]. For example, *Arabidopsis thaliana* contains 21 HSF genes, while rice (*Oryza sativa*) and Sorghum (*Sorghum bicolor*) possess 25 members [15,35]. Large gene families are also observed in soybean (52 HSFs) and wheat (56 HSFs), reflecting the evolutionary complexity and adaptive flexibility of HSF networks in polyploid crops [15,36]. Evolutionary expansion is closely tied to genome duplication events and environmental adaptation. Comparative genomic studies reveal that HSF clusters often align with loci under positive selection in response to thermal or drought stress [37,38]. Certain subfamilies, such as HSFA1 and HSFA2, are widely conserved as core regulators across species, while others exhibit lineage-specific diversification linked to unique ecological pressures. This evolutionary plasticity underlies the capacity of HSFs to orchestrate complex stress regulatory networks fine-tuned to diverse plant habitats.

### 2.2. Transcriptional, Post-Transcriptional, Translational and Post-Translational Regulation

The expression of HSF genes themselves is regulated at multiple levels and tightly controlled by developmental cues and environmental signals [13,39]. Many HSFs are induced transcriptionally upon heat, drought, oxidative, or hormonal stimuli, often mediated by upstream transcription factors such as DREB2A, WRKY, or bZIP proteins [40,41,42]. HSFA1 proteins, considered master regulators, can activate downstream HSFs like HSFA2, HSFA3, and HSFB1, forming a hierarchical cascade that amplifies stress responses [19,22,43]. The post-transcriptional regulation of plant HSFs is complex and involves alternative splicing [39,44]. For example, in Arabidopsis, a heat stress-induced HSFA2 variant, HSFA2-III, encodes a truncated isoform (S-HSFA2) that auto-activates its transcription via exon skipping in the DNA-binding domain (DBD) intron [45]. In rice, OsHSFA2d produces two variants: the inactive OsHSFA2dII (dominant under normal conditions, nuclear/cytoplasmic) and the active OsHSFA2dI (nuclear, heat stress-induced), which regulates heat stress response [46]. These examples highlight diversified post-transcriptional HSF regulation in plants.

The activity of HSFs is tightly regulated by post-translational modifications (PTMs), including phosphorylation, SUMOylation, acetylation, and ubiquitination, which collectively fine-tune their stability, localization, and transcriptional capacity [13,15,47,48]. Phosphorylation is the most extensively characterized PTM and often serves as an activating switch. For instance, MAPK-mediated phosphorylation of HSFA2 regulates its nuclear shuttling and transcriptional activity during heat and oxidative stress [49,50]. In contrast, SUMOylation generally acts as a repressive modification that limits HSF transcriptional activity or promotes interaction with negative regulators, ensuring attenuation of the stress response once stress subsides [51]. Notably, some cases, such as SUMOylation of TaHsfA1 in wheat, can enhance transcriptional activation, indicating functional diversity across species [52].

Acetylation and ubiquitination provide additional layers of control that balance HSF activation and turnover. Acetylation of lysine residues, catalyzed by histone acetyltransferases like HAC1, enhances HSF DNA-binding affinity and transactivation potential, reinforcing strong transcriptional responses and contributing to epigenetic memory through histone acetylation at HSP promoters [53,54]. Conversely, ubiquitination targets inactive or excess HSFs for degradation via the 26S proteasome, preventing sustained activation and restoring homeostasis after stress [55]. Together, these PTMs interact in a sequential or antagonistic manner, with phosphorylation and acetylation activating, SUMOylation repressing, and ubiquitination terminating, to ensure precise temporal and spatial regulation of HSF function within the broader plant stress signaling network.

Notably, the expression of HSFs is also modulated through liquid–liquid phase separation (LLPS). Stress granules (SGs), a major type of biomolecular condensate formed under heat stress, are conserved cytoplasmic messenger ribonucleoprotein assemblies that arise from pools of mRNAs stalled at translation initiation and incorporate translation initiation factors along with diverse RBPs and non-RBPs [56]. Because HSF expression is rapidly induced during heat stress to promote thermotolerance, maintaining the stability of their transcripts is crucial. In *Arabidopsis thaliana*, heat stress triggers the LLPS-driven condensation of ALBA4/5/6 proteins into SGs, where they protect HSF mRNAs from degradation by the exoribonuclease XRN4. Likewise, the glycine-rich RNA-binding proteins RBGD2 and RBGD4 undergo LLPS to form SGs that safeguard key heat-responsive transcripts, including HSFA2 and HSP70, thereby enhancing thermotolerance [56].

### 2.3. Subcellular Localization and Oligomerization Dynamics

HSFs are synthesized as inactive monomers that shuttle between the cytoplasm and nucleus. Under non-stress conditions, they are maintained in an inactive state through interactions with HSP70/HSP90 chaperone complexes. Upon stress exposure, unfolded proteins accumulate and titrate away these chaperones, releasing HSFs to trimerize, translocate to the nucleus, and bind HSEs [43,57]. After stress relief, feedback mechanisms mediated by HSPs reestablish homeostasis by promoting HSF monomerization and cytoplasmic sequestration. This dynamic nucleocytoplasmic cycling ensures that HSF activation is transient and tightly controlled, preventing unnecessary metabolic costs during normal growth. Importantly, differences in nuclear localization signals (NLS) and nuclear export signals (NES) among HSF classes contribute to functional specialization and temporal regulation of gene expression during successive stress phases [12,19].

In summary, the structural modularity and multilayered regulation of HSFs confer remarkable flexibility and specificity, enabling them to act as both general and specialized regulators of plant stress responses. The integration of transcriptional, post-transcriptional, and post-translational control allows plants to rapidly fine-tune HSF activity in response to environmental fluctuations. These foundational mechanisms underpin the canonical and non-canonical roles of HSFs discussed in subsequent sections.

## 3. Canonical Function of HSFs in Heat Stress Response

Heat stress is one of the most pervasive environmental threats affecting plant survival, growth, and productivity [2,58]. Elevated temperatures disrupt protein folding, destabilize membranes, and impair photosynthetic machinery, leading to the accumulation of misfolded or denatured proteins and reactive oxygen species (ROS) [59,60]. To counter these effects, plants have evolved a well-orchestrated heat stress response (HSR), a complex regulatory network that restores cellular homeostasis through the induction of heat shock proteins (HSPs) and other protective molecules [61]. At the transcriptional level, the HSR is primarily governed by heat shock transcription factors (HSFs), which act as the master regulators of heat-induced gene expression [15,62]. Activation of HSFs triggers a rapid and massive transcriptional reprogramming, ensuring timely expression of HSPs, antioxidant enzymes, and signaling mediators that collectively confer thermotolerance.

### 3.1. Activation and Dynamics of HSFs During Heat Stress

Under normal conditions, HSFs exist in the cytoplasm as inactive monomers, repressed by direct association with HSP70 and HSP90 chaperone complexes [39,63,64]. Heat-induced protein unfolding sequesters these chaperones, freeing HSF monomers to undergo trimerization via their HR-A/B domains, a critical step for activation [15]. The resulting HSF trimers translocate to the nucleus, where they bind specifically to heat shock elements (HSEs) in promoter regions of target genes, initiating transcription [19]. This activation is rapid and reversible, reflecting an efficient feedback system that balances protective gene expression with energy conservation. Once cellular proteostasis is restored, newly synthesized HSPs rebind to active HSFs, promoting their monomerization and cytoplasmic sequestration, thereby terminating the heat response. Such autoregulatory loops ensure that thermotolerance mechanisms are transient and tightly modulated.

### 3.2. HSFA1 as the Master Regulator

**Figure 2:**
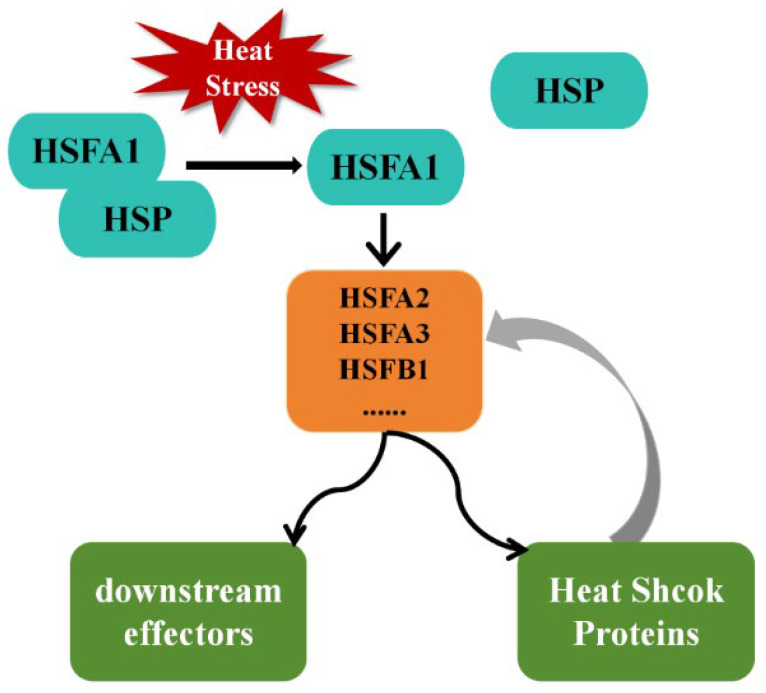
Hierarchical activation cascade of HSFs during heat stress response. Under non-stress conditions, HSFs are repressed by HSP70/HSP90 complexes. Heat-induced protein unfolding releases HSFA1, which trimerizes, translocates to the nucleus, and activates secondary HSFs (HSFA2, HSFA3, HSFB1) and downstream effectors such as HSPs and antioxidant enzymes. Newly synthesized HSPs feed back to repress HSFs, maintaining proteostasis.

Among plant HSFs, HSFA1 is widely recognized as the master regulator of the heat stress response [22,52]. Arabidopsis mutants lacking HSFA1 isoforms (HSFA1a, HSFA1b, HSFA1d, HSFA1e) exhibit dramatically reduced thermotolerance and fail to activate downstream HSFs and HSPs. HSFA1 directly binds to HSEs in promoters of HSFA2, HSFA3, HSFB1, and MBF1C, initiating a hierarchical transcriptional cascade that amplifies and sustains the heat response (Figure 2) [22].

HSFA1 also interacts with signaling proteins such as DEHYDRATION-RESPONSIVE ELEMENT BINDING PROTEIN 2A (DREB2A) and NAC transcription factors, forming regulatory hubs that link heat stress perception to broader transcriptional reprogramming [13,62]. Furthermore, HSFA1 is modulated by post-translational modifications such as phosphorylation and sumoylation, which fine-tune its activity in response to the intensity and duration of heat stress [13,65].

### 3.3. HSFA2 as the Amplifier of Sustained Thermotolerance

While HSFA1 triggers the initial HSR, HSFA2 functions as a potent amplifier of sustained thermotolerance. HSFA2 expression is strongly induced by heat, oxidative stress, and certain phytohormones, and its protein accumulates rapidly during prolonged or repeated stress exposure [66]. Acting downstream of HSFA1, HSFA2 activates a large subset of heat-responsive genes, which encode HSPs, chaperonins, and redox-regulating enzymes such as ascorbate peroxidases (APXs) and glutathione *S*-transferases (GSTs) [57,67,68].

HSFA2 also contributes to transcriptional memory, the phenomenon whereby plants exhibit enhanced tolerance to recurring heat stress. Upon a second heat episode, previously activated HSFA2 rebinds rapidly to HSEs, often in cooperation with histone modifications (e.g., H3K4me3) that maintain chromatin in a primed state [24,26]. This molecular memory allows plants to recall previous stress encounters and mount faster, stronger responses, a key feature of adaptive thermotolerance.

### 3.4. Roles of Other HSF Classes in Heat Response

In addition to HSFA1 and HSFA2, several other HSFs participate in fine-tuning the HSR. HSFA3 is induced by the DREB2A-dependent pathway and contributes to the long-term acclimation phase of heat stress by regulating genes involved in osmoprotection and metabolism [42]. HSFA6b mediates ABA-responsive transcriptional activation under heat and drought stress, integrating hormonal and thermal cues [69,70]. HSFB1 and HSFB2b, though lacking classical activation domains, act as co-regulators, serving as either repressors or activators, depending on their interaction partners. HSFB1, for instance, interacts with and represses HSFA1 activity to modulate gene expression. Interestingly, in tomato, HSFB1 exhibits dual functions, serving as both a transcriptional repressor and a co-activator [15,71,72]. Such hierarchical and cooperative regulation allows plants to orchestrate a temporally layered HSR. Early-acting HSFs (e.g., HSFA1) initiate rapid protective gene expression, while late-acting ones (e.g., HSFA2, HSFA3) sustain or modulate responses according to stress duration and severity.

### 3.5. Heat Shock Proteins (HSPs) as Downstream Targets of HSFs

The principal downstream targets of HSFs are heat shock proteins (HSPs), a diverse family of molecular chaperones categorized into major classes, consisting of HSP100, HSP90, HSP70, HSP60, and small HSPs (sHSPs), based on molecular weight and function [73]. HSPs prevent protein aggregation, refold denatured proteins, and facilitate the degradation of irreversibly damaged polypeptides [74]. For instance, HSP70 and HSP90 act as central hubs of proteostasis, directly interacting with signaling proteins and HSFs to regulate activity and recovery phases. sHSPs, localized in various organelles (chloroplasts, mitochondria, peroxisomes), stabilize thylakoid membranes and photosystem II during heat stress, thereby maintaining photosynthetic efficiency [75]. HSP100 (ClpB) family members, such as HSP101, are required for thermotolerance by resolubilizing aggregated proteins, a process essential for post-stress recovery [76]. The tight feedback between HSFs and HSPs ensures a dynamic equilibrium. HSFs activate HSP expression, while HSPs, in turn, repress excessive HSF activity, constituting a self-regulating chaperone circuit critical for cellular balance.

### 3.6. Coordination with ROS and Antioxidant Systems

Heat stress invariably perturbs redox homeostasis, leading to elevated ROS production. HSFs play a pivotal role in linking ROS signaling with transcriptional regulation. For example, HSFA2 and HSFA4a activate genes encoding ascorbate peroxidase (APX), superoxide dismutase (SOD), and thioredoxin enzymes that detoxify ROS and mitigate oxidative damage [77,78,79]. Moreover, ROS themselves can act as secondary messengers that modulate HSF activation through redox-sensitive cysteine residues, establishing a feedback mechanism that couples oxidative status with transcriptional control [13,80].

### 3.7. Heat Stress Memory and Epigenetic Regulation

Recent studies have shown that plants possess a form of heat stress memory, whereby exposure to a mild heat stress primes the genome for quicker reactivation upon subsequent stress [81]. HSFs, particularly HSFA2, are central to this process. After initial activation, HSFA2 associates with chromatin remodelers and histone modifiers, maintaining a histone methylation landscape (H3K4me3, H3K9ac) at promoters of heat-responsive genes [53,82]. This epigenetic imprinting ensures transcriptional readiness and contributes to acquired thermotolerance, a hallmark of plant stress adaptation.

In summary, the canonical role of HSFs in the heat stress response exemplifies a highly organized regulatory hierarchy. HSFA1 functions as the master switch initiating the response, HSFA2 sustains and amplifies it, and other HSFs modulate fine-tuning, integration, and feedback. Together, these factors orchestrate a comprehensive protective system encompassing chaperone induction, redox regulation, and epigenetic memory formation. The mechanistic complexity and versatility of HSFs in thermotolerance provide the foundation for their extended roles in other environmental stress contexts, as explored in the next section.

## 4. Multifunctional Roles of HSFs in Other Abiotic Stresses

Although initially identified as key regulators of thermotolerance, accumulating evidence reveals that HSFs are versatile players in plant adaptation to a wide spectrum of abiotic stresses, including drought, salinity, cold, oxidative stress, and heavy metal toxicity [10,11,12,13]. This multifunctionality arises from the broad activation mechanisms of HSFs and their integration with hormonal and redox signaling pathways that respond to diverse stress cues. The ability of HSFs to sense and interpret multiple stress signals makes them central integrators within the broader plant stress regulatory network. In many cases, distinct HSF isoforms are activated not only by heat but also by drought, high salinity, or cold exposure, leading to overlapping transcriptional outputs that promote cross-tolerance, a phenomenon where exposure to one stress enhances resistance to another.

### 4.1. HSFs in Drought Stress Tolerance

Drought stress induces cellular dehydration, osmotic imbalance, and oxidative damage, activating transcriptional programs that overlap substantially with the heat stress response. Several HSF genes are upregulated under water deficit and play crucial roles in enhancing drought tolerance through coordination with the abscisic acid signaling pathway. In Arabidopsis, HSFA6a and HSFA6b function as ABA-dependent transcriptional activators. *HSFA6a* expression is rapidly induced by ABA and dehydration, and its overexpression enhances drought tolerance via upregulation of *LEA* proteins, HSPs, and ROS-scavenging enzymes [69]. HSFA6 binds directly to ABA-responsive elements (ABREs) in target gene promoters and cooperates with bZIP transcription factors such as ABF3/4, integrating HSF and ABA signaling [22]. Homologous genes in wheat (*TaHSFA6f*) and maize (*ZmHSFA6b*) exhibit similar regulatory patterns, suggesting evolutionary conservation across plant lineages [36].

Beyond its classical role in thermotolerance, HSFA2 contributes to drought resilience by maintaining protein homeostasis and redox balance. It activates cytosolic and chloroplastic HSPs, APX1, and GSTU17, thereby reducing oxidative damage associated with water deficit [83,84]. Overexpression of *HSFA2* in tomato and rice enhances drought recovery and preserves photosynthetic efficiency [39]. Other HSF family members also participate in drought adaptation. *HSFA1a* and *HSFA1b* overexpression improves water utilization and drought resistance in tomato and Arabidopsis [85,86], while HaHSFA4a and HaHSFA9 from sunflower confer enhanced tolerance in transgenic tobacco [87]. Members of the HSFB and HSFC classes serve as fine-tuners of drought response. HSFB1 interacts with HSFA1 and MBF1C to regulate drought-inducible chaperones [15], whereas HSFC1 adjusts stress gene expression in rice and maize. Interestingly, HSFBs can exert both positive and negative regulatory effects. ZmHSF08 and OsHSFB2b act as repressors of drought tolerance in maize and rice [88], while HvHSFB2c enhances tolerance in barley [89]. Likewise, wheat *TaHSFC2a-B* mediates combined drought and heat resilience during grain filling through ABA-dependent mechanisms. Collectively, these studies demonstrate that HSFs act as central integrators of drought signaling, functioning in concert with ABA and redox networks to preserve cellular homeostasis and sustain growth under dehydration stress.

### 4.2. HSFs in Salinity Stress Adaptation

High salinity imposes osmotic and ionic stress, disrupting homeostasis and redox balance. HSFs enhance salt tolerance by activating osmoprotectant biosynthesis, ion transport, and antioxidant defense. In *Arabidopsis*, *HSFA1a* and *HSFA2* are upregulated under salt stress, inducing *HSP70*, *HSP101*, and *APX1* to mitigate oxidative damage [23,90]. *hsfa1* mutants exhibit NaCl hypersensitivity and ROS accumulation, whereas *HSFA1* overexpression restores redox balance and ion selectivity [22]. HSFs also intersect with the SOS and ABA signaling pathways. *HSFA6a* interacts with ABF/AREB transcription factors to activate *SOS1*, *NHX1*, and *P5CS*, linking ion homeostasis and osmotic protection [36,69]. In rice, OsHSFA2e and OsHSFA4d mediate salt-induced transcriptional memory via persistent histone acetylation, facilitating rapid gene reactivation during recurrent stress [91].

Diverse HSF family members contribute differentially to salinity tolerance. Overexpression of *OsHSFA7* enhances salt resistance in rice [92], whereas tomato *SlHSFA3* increases Arabidopsis salt sensitivity [93]. A wheat HSF TaHSFA2d improves germination and tolerance in Arabidopsis under salt stress [94], and HSFA4a deficiency heightens salt sensitivity [95]. Among Arabidopsis HSFs, HSFA6b shows particularly strong induction under salinity [96]. HSFB and HSFC members also participate in salt regulation. GmHSFB2b in soybean promotes flavonoid synthesis and ROS detoxification, thereby enhancing salt tolerance [97], while ZmHSF08 in maize and OsHSFB2b in rice act as negative regulators, with overexpressing lines exhibiting increased salt sensitivity [88,98]. Collectively, HSFs integrate ionic, osmotic, and oxidative stress responses, functioning as central transcriptional and epigenetic regulators that shape plant resilience under salinity.

### 4.3. HSFs in Cold Stress Tolerance

Cold stress disrupts membrane fluidity and cellular metabolism, triggering extensive transcriptional reprogramming [99,100]. Although primarily associated with thermotolerance, some HSFs have emerged as crucial mediators of cold acclimation and chilling tolerance. HSFA1 not only governs heat stress responses but also modulates cold signaling by activating CBF/DREB1 transcription factors, central regulators of cold-responsive genes [69,101]. Transcriptome analyses have revealed significant upregulation of several HSFs in forage crops exposed to cold stress, including *MsHSF04*, *MsHSF11*, and *MsHSF13* in alfalfa (*Medicago sativa*) [102], as well as *BdHSF09*, *BdHSF14*, *BdHSF20*, and *BdHSF22* in *Brachypodium distachyon* [103]. In maize (*Zea mays*), *HSF21* enhances cold tolerance by regulating lipid metabolism genes such as *GPATs* and *SADs*, while knockout mutants exhibit reduced tolerance [104]. In rice (*Oryza sativa*), *OsHSFA3*, *OsHSFA4d*, and *OsHSFA9* are induced by cold stress, whereas most *HSFB*-like genes are repressed except *OsHSFB4a* and *OsHSFB4b*. Members of the HSFC family, especially OsHSFC1, are strongly activated, suggesting a role in low-temperature sensing [105]. Similar induction occurs in carrot (*Daucus carota*), where *DcHSF16*, belonging to HSFC family, responds to cold stress [106]. In banana (*Musa acuminata*), MaHSF24 acts as a negative regulator of chilling tolerance by repressing *MaHSP23.6/70* and antioxidant genes (*MaAPX1*, *MaGSTZ1*) [107]. Together, these findings highlight HSFs as key integrators of cold stress responses, coordinating transcriptional, metabolic, and redox adjustments for improved plant resilience.

### 4.4. HSFs in Heavy Metal Stress Tolerance

HSFs are also involved in plant defense against heavy metal stress (e.g., Cd), mainly by activating detoxification and antioxidant pathways. In wheat, HsfA4a confers Cd tolerance by upregulating metallothionein gene expression [108]. In the Cd/Zn/Pb hyperaccumulator *Sedum alfredii*, 22 *HSF* genes were identified, with 18 members showing Cd-responsive expression [109]. Among them, *SaHSFA4c* transcripts and proteins were strongly induced by Cd in all tissues, leading to the activation of ROS-related genes and HSPs, which in turn reduced ROS accumulation in transgenic *Arabidopsis* and in the non-hyperaccumulating ecotype of *S. alfredii* [110]. Transcriptomic studies in Cd-treated switchgrass roots further revealed that HSF/HSP modules contribute to maintaining protein homeostasis and proper folding under Cd stress, while overexpression of an *HSP* gene in *Arabidopsis* markedly improved Cd tolerance [111]. In tomato, HsfA1a promotes Cd tolerance by inducing melatonin biosynthesis [112]. Likewise, *PuHSFA4a* from *Populus ussuriensis* activates target genes *PuGSTU17* and *PuPLA*, enhancing antioxidant defenses and root development to support Zn tolerance [113]. Collectively, class A HSFs enhance heavy metal tolerance by regulating genes associated with metal chelation, ROS scavenging, and cellular homeostasis.

In summary, HSFs constitute a multifunctional transcription factor family that transcends their classical role in thermotolerance. Through diverse isoforms, post-translational modifications, and signaling crosstalk, they orchestrate complex defense networks against drought, salinity, cold, oxidative, and toxic stresses (Table 1). The evolutionary expansion and diversification of HSFs have allowed plants to build robust and flexible stress response systems, underpinning the concept of HSFs as universal stress integrators.

## 5. HSFs in Biotic Stress Responses

### 5.1. Expanding the Functional Landscape: From Abiotic to Biotic Stress

While HSFs are best known for their roles in abiotic stress responses, mounting evidence indicates their significant contribution to biotic stress resistance [19]. Biotic and abiotic stress signaling pathways often converge at the transcriptional level, with HSFs acting as key integrators of these combined responses.

Pathogen infection triggers massive transcriptional reprogramming, activating defense-related genes, secondary metabolism, and hormone signaling pathways [123]. Intriguingly, several HSFs are induced not only by temperature or drought but also by bacterial, fungal, and viral attacks, indicating a shared regulatory framework between thermal and immune responses [11,124,125,126]. This dual functionality suggests that HSFs evolved as versatile regulatory hubs that link abiotic and biotic responses.

### 5.2. Role of HSFs in Plant Pathogen Defense

Plants rely on a multilayered immune system, comprising pattern-triggered immunity (PTI) and effector-triggered immunity (ETI), to defend against pathogen invasion. Recent studies have demonstrated that HSFs actively participate in these immune processes by modulating defense-related transcriptional networks, reactive oxygen species (ROS) homeostasis, and hormone-mediated signaling pathways. In *Arabidopsis thaliana*, several HSFs are rapidly induced upon pathogen attack. HSFA1a, HSFA2, and HSFA4a are activated following infection by *Pseudomonas syringae*, contributing to disease resistance through the upregulation of pathogenesis-related (PR) genes [85,127]. Overexpression of *HSFA1a* enhances salicylic acid (SA)-dependent defense and systemic acquired resistance (SAR), whereas *AtHSFA2* functions in the full activation of SA-mediated immune responses [128,129]. In contrast, HSFB1 and HSFB2b, typically acting as repressors of the heat shock response, exert complex regulatory roles in immunity [23]. HSFB1 negatively modulates jasmonic acid (JA)- and ethylene-responsive defensin genes, which affects resistance against the necrotrophic fungus *Alternaria brassicicola* [124]. Moreover, *HSFB1* expression is induced by SA and mediates the balance between growth and defense [130], suggesting its involvement in resource allocation under biotic stress.

Functional studies in other plant species further highlight the conserved and diversified roles of HSFs in pathogen defense. In pepper (*Capsicum annuum*), silencing of *CaHSFB2a* compromises resistance to *Ralstonia solanacearum*, while its overexpression triggers hypersensitive response (HR)-like cell death, H_2_O_2_ accumulation, and activation of defense marker genes, establishing a positive feedback loop with *CaWRKY6* and *CaWRKY40* [131]. Similarly, overexpression of *OsHSFB4d* in rice enhances resistance to *Xanthomonas oryzae* pathovars, accompanied by elevated expression of *OsHsp18.0-CI* and multiple PR genes [121].

HSFs also contribute to pathogen resistance through integration with hormone signaling and redox regulation. In rice, OsHSFA2d activates JASMONATE ZIM-DOMAIN (JAZ) genes under heat stress, linking the HSF pathway to JA-mediated defense, while AtHSFA1b in *Arabidopsis* promotes JA biosynthesis via activation of *AtOPR3* [132,133]. In barley, overexpression of *HvHSFA2e* confers broad-spectrum stress tolerance through modulation of hormone balance and secondary metabolism [134]. In cassava (*Manihot esculenta*), *MeHSF3* directly binds to the promoters of *EDS1* and *PR4*, reinforcing defense against *Xanthomonas axonopodis* pv. *Manihotis* [135]. Likewise, in tomato, *HSFA1a* regulates *RBOH*-dependent ROS production through the HSFA1a–Wfi1 signaling cascade, initiating an early oxidative burst during infection by *Meloidogyne incognita* [136].

Collectively, these findings establish HSFs as integral regulators of plant immunity that interface with classical defense hormones, redox signaling, and transcriptional control. By orchestrating the expression of defense-related genes and mediating crosstalk among SA, JA, and ROS pathways, HSFs function not only as stress-responsive transcription factors but also as key modulators linking environmental and biotic stress resilience.

## 6. Crosstalk Between HSFs and Phytohormones

Plant stress responses are not isolated linear pathways but interconnected signaling networks integrating hormonal, metabolic, and redox cues [137,138,139]. Heat shock transcription factors (HSFs) lie at the intersection of these networks, translating upstream signals into precise transcriptional outputs that promote stress adaptation. Recent studies demonstrate that HSFs interact with phytohormone pathways such as abscisic acid (ABA), ethylene (ET), salicylic acid (SA), jasmonic acid (JA), and auxin [12,19]. This crosstalk allows plants to coordinate local and systemic responses, balance growth and defense, and fine-tune stress tolerance depending on developmental stage and stress duration.

### 6.1. Crosstalk Between HSFs and Abscisic Acid (ABA) Signaling

ABA is a pivotal hormone coordinating plant adaptation to abiotic stresses such as drought, salinity, and temperature extremes. Increasing evidence demonstrates that heat shock transcription factors (HSFs) are deeply integrated into ABA-mediated stress signaling networks, forming reciprocal regulatory loops that enhance plant resilience.

In *Arabidopsis*, the expression of *HSFA6b* is induced by ABA through the action of ABA-RESPONSIVE ELEMENT-BINDING PROTEIN1 (AREB1), a key transcriptional activator in ABA signaling [69]. Once activated, HSFA6b promotes the transcription of *DREB2A* (DEHYDRATION-RESPONSIVE ELEMENT-BINDING PROTEIN2A), thereby bridging the ABA and heat shock regulatory pathways. Thus, HSFA6b functions as a positive regulator not only of thermotolerance but also of drought and salinity tolerance. Moreover, HSFA6b participates in multiple ABA-dependent developmental and physiological processes, including seed germination, cotyledon greening, and root growth, highlighting its broad regulatory scope. Other HSF members are similarly integrated within the ABA network. During seed maturation, the ABA pathway component ABI3 regulates *HSFA9* expression in *Arabidopsis*, linking HSF activity to desiccation tolerance during seed development [40]. In crops, this crosstalk is evolutionarily conserved. For instance, in wheat (*Triticum aestivum*), three homologs of *TaHSFC2a* are induced by ABA, heat, and drought stress, with *TaHSFC2a-B* enhancing heat tolerance during grain filling through an ABA-mediated pathway [122]. Likewise, in rice (*Oryza sativa*), OsHSFA3 enhances drought tolerance by elevating endogenous ABA levels and boosting antioxidant capacity [140]. Collectively, these findings underscore the close interplay between HSFs and ABA signaling. Through mutual regulation, ABA-responsive HSFs serve as crucial integrators of hormonal and environmental signals, fine-tuning transcriptional networks that govern plant stress tolerance and developmental plasticity.

### 6.2. Crosstalk Between HSFs and Ethylene Signaling

Ethylene signaling plays a vital role in plant thermotolerance by mitigating heat-induced oxidative damage [141]. Recent studies reveal that HSFs and ethylene signaling components are functionally interconnected through a shared transcriptional network. In *Arabidopsis*, activation of the ethylene pathway triggers a cascade mediated by ethylene response factor (ERF) transcription factors. Notably, ERF95 and ERF97 directly bind to the promoter of *HSFA2*, thereby inducing its expression and establishing a mechanistic link between ethylene signaling and the heat shock response [142]. Furthermore, HSFA7b functions as a key regulator of thermomemory in the *Arabidopsis* shoot apical meristem by modulating the expression of ethylene biosynthesis and signaling genes, including *ETHYLENE INSENSITIVE 3* (*EIN3*) [143]. This reciprocal regulation underscores the bidirectional nature of HSF–ethylene interactions, where ethylene signaling activates specific HSFs, and in turn, HSFs fine-tune ethylene-mediated responses.

The relevance of this crosstalk extends beyond *Arabidopsis*. The root endophyte Enterobacter sp. SA187 enhances thermotolerance in both *Arabidopsis* and wheat through ethylene-mediated signaling and by inducing thermomemory-associated histone modifications [144]. In rice (*Oryza sativa*), multiple HSFs, including OsHSFA1a, OsHSFA2a, OsHSFA2c, OsHSFA2d, OsHSFA2e, and OsHSFA2f, contribute to heat tolerance by integrating into the ethylene signaling pathway, further supporting the conserved role of HSF–ethylene interplay in orchestrating heat resilience across plant species [145]. Together, these findings highlight ethylene as a key hormonal partner of HSFs in coordinating thermotolerance and thermomemory through transcriptional and epigenetic mechanisms.

### 6.3. Crosstalk Between HSFs and Brassinosteroid Signaling

Brassinosteroids (BRs) are essential plant steroid hormones best known for promoting growth, development, and stress adaptation through a phosphorylation-dependent signaling cascade. Upon BR perception by the receptor complex containing BRASSINOSTEROID INSENSITIVE 1 (BRI1), a signaling module involving BR INSENSITIVE 2 (BIN2) kinase and BRI1 SUPPRESSOR 1 (BSU1) phosphatase regulates the activation of the transcription factors BRI1-EMS-SUPPRESSOR 1/BRASSINAZOLE-RESISTANT 2 (BES1/BZR2) and BZR1 via dephosphorylation [146]. Beyond their developmental roles, BRs play a crucial part in thermotolerance. Recent advances have clarified the molecular mechanisms linking BR signaling to the heat shock response via direct interaction with HSFs [61]. Heat stress induces dephosphorylation and nuclear accumulation of BES1, which cooperates with HSFA1a at heat shock elements to activate *HSP70* and *HSP90* gene expression [147,148]. Functional analyses further confirm this regulatory role. BES1 gain-of-function mutants display enhanced heat tolerance, whereas BES1 loss-of-function mutants exhibit reduced thermotolerance [149,150]. Similarly, a dominant mutation in BZR1 (*bzr1-D*) confers elevated heat stress resistance [151], suggesting a conserved role for BZR-like proteins in BR–HSF crosstalk.

The interplay between BR signaling components and HSFs appears to involve multiple regulatory layers. For example, heat-induced BES1 activation occurs independently of BRI1, as demonstrated in the *bri1-1* mutant [150]. However, a different *bri1* allele (*bri1-116*) shows decreased survival under heat stress [152], indicating potential context-dependent contributions of BRI1 to thermotolerance. In contrast, BIN2 that represses BES1 also phosphorylates HSFA1d, preventing its nuclear localization and transcriptional activity during the HSR [153]. Intriguingly, both BES1 and BIN2 are clients of HSP90, which regulates their nuclear translocation and stability [154,155]. This points to a potential feedback loop wherein the HSR modulates BR signaling components to fine-tune stress recovery and post-stress growth. Collectively, these findings highlight a sophisticated network of HSF–BR interactions, where BR-responsive transcription factors such as BES1 and BZR1 cooperate with HSFs to activate HSP expression, while kinases like BIN2 dynamically balance stress signaling and developmental growth.

### 6.4. Crosstalk Between HSFs and Auxin, Jasmonic Acid, and Salicylic Acid Signaling

Emerging evidence reveals multifaceted crosstalk between HSFs and phytohormones such as auxin, jasmonic acid (JA), and salicylic acid (SA), highlighting their roles as central integrators of stress and hormonal responses [19]. Auxin signaling modulates root architecture and stress adaptation, in part through interactions with HSFs. In poplar (*Populus trichocarpa*), the auxin repressor IAA17.1 physically interacts with HSFA5a, a root-enriched and salt-inducible transcription factor [156]. Overexpression of *HSFA5a* enhances flavonol accumulation, reduces reactive oxygen species (ROS) buildup, and promotes lateral root growth and salt tolerance, suggesting that auxin–HSF interplay fine-tunes redox balance and developmental plasticity under stress. Similarly, in sunflower (*Helianthus annuus*), HaIAA27 interacts with HaHSFA9, repressing its transcriptional activity [157]. These findings indicate that Aux/IAA proteins can directly modulate HSF function, linking auxin homeostasis with HSF-mediated stress regulation.

The interaction between HSFs and JA signaling contributes to both thermotolerance and defense responses. In Arabidopsis, HSFB1 and HSFB2b act as repressors of the heat shock response but positively regulate acquired thermotolerance [158]. Intriguingly, HSFB1 also functions as a negative regulator of *PDF1.2a* and *PDF1.2b*, two JA-responsive defensin genes, thereby modulating innate immunity against necrotrophic pathogens such as *Alternaria brassicicola* [124]. Beyond Arabidopsis, TaHSFA1b in wheat enhances heat tolerance through JA-mediated signaling pathways [133], while in rice (*Oryza sativa*), HTG3/OsHSFA2d activates JASMONATE ZIM-DOMAIN (JAZ) genes under heat stress, reinforcing the connection between HSF activity and JA-responsive transcription [159]. Similarly, HSFA1b in Arabidopsis induces AtOPR3, a key enzyme in JA biosynthesis, establishing a positive regulatory loop between JA production and HSF activity [133]. In barley (*Hordeum vulgare*), HvHSFA2e has been shown to confer multi-stress tolerance by modulating hormonal signaling and secondary metabolism [134].

HSFs also participate in SA-dependent defense and thermotolerance mechanisms. In Arabidopsis, *HSFB1* is induced by SA and mediates trade-offs between growth and defense, with reduced expression of *HSFB1* in F1 hybrids correlating with enhanced biomass accumulation [130]. In pepper (*Capsicum annuum*), silencing of *CaHSFB2a* compromises resistance to *Ralstonia solanacearum*, whereas its overexpression triggers hypersensitive response (HR)-like cell death, H_2_O_2_ accumulation, and activation of SA-related defense genes [131]. Furthermore, in tomato (*Solanum lycopersicum*), SA treatment under heat stress enhances HSF expression and DNA-binding activity, upregulating HSP70 and improving thermotolerance [128].

In summary, HSFs occupy a central regulatory position at the crossroads of phytohormones. They are simultaneously activated by and regulate hormones such as ABA, ET, BR, SA, JA, and auxin. Through these interactions, HSFs orchestrate dynamic feedback loops that harmonize growth, defense, and metabolic stability. This intricate crosstalk underscores the conceptual shift from viewing HSFs as mere heat-response regulators to recognizing them as core integrators of plant stress signaling networks.

## 7. Applications

The growing understanding of heat shock transcription factors (HSFs) as central integrators of plant stress responses has opened new opportunities for biotechnological innovation and crop improvement. By manipulating HSF regulatory networks, we can potentially enhance multi-stress resilience, yield stability, and stress memory in plants, which are critical goals in the face of accelerating climate change and resource scarcity. This section discusses current translational applications, biotechnological strategies, and emerging research directions that could harness HSFs for agricultural and ecological benefits.

### 7.1. Engineering HSFs for Multi-Stress Tolerance

Early attempts to engineer thermotolerance focused on overexpressing single HSF genes, often resulting in enhanced heat tolerance. For example, overexpression of HSFA1a or HSFA2 in Arabidopsis confers improved survival under heat stress by upregulating chaperone and antioxidant genes [79]. Similarly, transgenic tomato plants overexpressing SlHSFA1a or SlHSFA2 exhibit increased tolerance not only to heat but also to drought and oxidative stress [39]. These results highlight the pleiotropic effects of HSFs that extend beyond their canonical thermo-protective role.

In rice (*Oryza sativa*), overexpression of *OsHSFA2e* and *OsHSFA3* enhances both heat and salt tolerance, accompanied by increased accumulation of osmoprotectants, such as proline and soluble sugars [92]. Similarly, maize (*Zea mays*) lines expressing *ZmHSFA2* or *ZmHSFB2b* show improved photosynthetic performance and yield stability under combined heat and drought stress. These studies demonstrate that HSFs represent valuable genetic targets for developing crops with broad-spectrum stress tolerance.

However, constitutive overexpression of HSFs may lead to growth penalties or altered developmental phenotypes due to the metabolic costs associated with persistent stress responses [62]. Therefore, fine-tuned expression systems using stress-inducible promoters (e.g., RD29A) or tissue-specific expression are recommended to minimize negative trade-offs. Synthetic biology approaches combining promoter engineering, inducible gene switches, and transcriptional feedback loops hold promise for achieving precision control of HSF activity.

### 7.2. Manipulating Downstream HSF Pathways

Beyond direct modification of HSF genes, engineering their downstream pathways offers another strategic route for enhancing resilience. Many HSFs regulate overlapping sets of heat shock proteins (HSPs), ROS scavengers, and osmoprotective enzymes, which act as effector modules of stress protection [15]. For example, manipulation of HSP70 or HSP101 under HSF promoter control has successfully improved thermotolerance in wheat and maize [94,116].

Recent advances in gene stacking and multiplex CRISPR editing enable simultaneous targeting of multiple HSF–HSP nodes, allowing synergistic enhancement of stress resistance without major yield penalties. Moreover, network-guided engineering based on transcriptomic co-expression data can identify key regulatory hubs within the HSF-centered interactome, guiding rational design of resilient genotypes.

In parallel, modulating HSF post-translational regulation represents an underexplored avenue. Small molecules or peptides that stabilize HSF–HSP interactions or alter HSF SUMOylation/ubiquitination status could offer chemical tools for stress management. For example, inhibition of specific E3 ubiquitin ligases that target HSFA2 increases thermotolerance in Arabidopsis. Future development of HSF-targeted regulators might thus complement genetic engineering for precise stress control.

### 7.3. Integrating HSF Networks with Hormonal and Epigenetic Engineering

Given that HSFs act at the crossroads of hormone signaling and epigenetic memory, their manipulation could be combined with broader network engineering approaches. ABA-inducible HSFs such as HSFA6A and HSFA6B are ideal entry points for enhancing drought and salinity tolerance through improved ABA responsiveness. Similarly, cross-regulation between HSFs and salicylic acid signaling components (e.g., NPR1) offers a route to integrate abiotic and biotic stress tolerance.

Recent findings that HSFs participate in chromatin remodeling and transgenerational stress memory raise exciting possibilities for epigenetic breeding. Plants exposed to repeated stress often display inherited tolerance traits associated with sustained HSF activity and histone modifications at HSF target genes [24]. Harnessing such stable epigenetic marks either through mild priming treatments or targeted epigenome editing (e.g., CRISPR-dCas9–TET/HAT fusions) could complement conventional breeding programs for climate-resilient crops.

### 7.4. HSFs as Biomarkers and Selection Tools in Breeding

Because HSFs are among the earliest and most responsive transcription factors induced under stress, they serve as reliable molecular biomarkers for screening stress-resilient genotypes. Transcript abundance of key HSFs such as HSFA1, HSFA2, and HSFB2b correlates strongly with thermotolerance and drought recovery in tomato, maize, and soybean [39]. High-throughput qPCR and RNA-seq profiling of HSF expression signatures can thus aid in marker-assisted selection (MAS) and genomic selection (GS) pipelines for breeding programs.

Furthermore, advances in machine learning and network inference allow predictive modeling of stress tolerance based on HSF-centered transcriptional modules. By integrating multi-omics datasets (transcriptomics, proteomics, metabolomics), breeders can identify robust biomarkers representing systems-level resilience rather than single-gene responses. Such integrative frameworks bridge molecular biology with quantitative genetics, accelerating the translation of HSF biology into field performance.

### 7.5. Limitations for Translational Use of HSFs

Despite promising results in greenhouses and controlled environments, several key bottlenecks impede the direct translation of heat shock factor (HSF) overexpression into field-level yield stability. Strong phenotypes observed in laboratory settings often diminish under complex field conditions, as HSF effects are highly context-dependent and generate substantial genotype × environment interactions. Constitutive or poorly regulated HSF overexpression frequently imposes growth and yield penalties due to metabolic burdens and resource reallocation, such as persistent chaperone synthesis and altered hormone balances, or by tilting the growth–defense equilibrium toward defense, thereby reducing biomass accumulation. Pleiotropy and redundancy within the HSF family further complicate predictable outcomes, as overexpressing one HSF may induce compensatory changes in other family members, hormone networks, or secondary metabolism, potentially heightening pathogen susceptibility or altering ecological interactions (e.g., with pollinators or soil microbes). Moreover, the epigenetic and transgenerational stability of engineered HSF states remains uncertain, with beneficial priming in one generation possibly resetting or leading to unintended carry-over effects in subsequent ones. Regulatory, biosafety, and public acceptance challenges also pose significant constraints, particularly for transgenic approaches in diverse agroecosystems.

Addressing these limitations will necessitate a multifaceted approach. Strategies include employing stress-inducible or tissue-specific promoters to prevent chronic activation; stacking complementary HSF/HSP and metabolic effector modules instead of relying on single-gene overexpression; conducting extensive multi-location and multi-year field trials to assess genotype × environment effects; integrating HSF modules into breeding pipelines via marker-assisted selection or genomic selection for adaptation to local backgrounds; and performing thorough ecological risk assessments. Prioritizing precision regulation and systems-level evaluation will be crucial to advance HSF engineering from promising proof-of-concept studies to robust, yield-stable agricultural applications.

## 8. Conclusions

**Figure 3:**
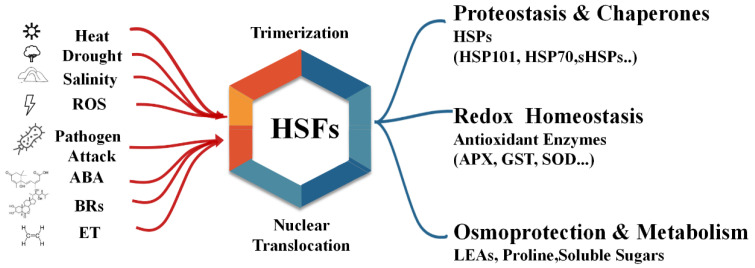
Schematic model illustrating the central role of HSFs in integrating a multitude of environmental and cellular signals to orchestrate broad-spectrum stress resilience in plants.

The accumulated body of evidence over the past decades has transformed our understanding of HSFs from specialized mediators of heat stress to global integrators of plant stress adaptation and resilience. Once regarded merely as activators of heat shock proteins, HSFs are now recognized as master regulators coordinating diverse molecular, physiological, and developmental processes in response to both abiotic and biotic cues (Figure 3). Their ability to sense perturbations in cellular proteostasis, integrate hormonal and redox signals, and imprint transcriptional memory positions them at the core of the plant’s adaptive network.

At the molecular level, HSFs exhibit remarkable versatility, shaped by domain modularity, post-translational modifications, and dynamic protein–protein interactions. These properties allow them to act as fine-tuned sensors and signal processors, linking external stress perception to genome-wide transcriptional reprogramming. Beyond thermotolerance, HSFs now stand at the crossroads of multi-stress responses, participating in the regulation of drought, salinity, cold, oxidative, and pathogen stresses through interconnected networks that confer robustness and plasticity.

From an evolutionary perspective, the expansion and diversification of HSF gene families across plant lineages illustrate adaptive innovation in the face of environmental complexity. Comparative genomics has revealed lineage-specific expansions in monocots and dicots, suggesting that HSF diversification parallels the ecological adaptation of plant species to distinct climates and habitats. Future exploration of these evolutionary trajectories could provide invaluable insights into the molecular basis of environmental resilience.

At the systems level, HSFs exemplify the principles of hierarchical regulation and network resilience. The HSF–HSP–proteostasis feedback loop functions as a molecular thermostat, maintaining cellular equilibrium under fluctuating conditions. Crosstalk between HSFs and hormonal, ROS, and epigenetic pathways forms a multilayered communication system that allows plants to integrate information across temporal and spatial scales. This capacity to balance immediate stress defense with long-term developmental priorities underpins the concept of multi-stress resilience, in which plants coordinate complex trade-offs to ensure survival and productivity.

The emerging frontiers of synthetic biology, genome editing, and systems modeling now offer unprecedented opportunities to harness HSF networks for sustainable agriculture. Through precision engineering, it is conceivable to construct plants with programmable stress responses, capable of dynamically adjusting to temperature fluctuations, drought, or salinity without compromising yield. The development of synthetic HSF circuits, stress-inducible promoters, and CRISPR-based regulatory modules can transform HSF biology into tangible agricultural technologies. Moreover, integrating epigenetic memory engineering with HSF manipulation may enable the design of crops capable of retaining adaptive “memories” across generations, opening new avenues for durable stress tolerance.

Nevertheless, several challenges persist. Unraveling the context dependency, redundancy, and cross-family interactions among HSFs remains an ongoing task. Future research must also consider the ecological and evolutionary implications of manipulating such central regulators, as excessive or mis-regulated activation could disrupt plant homeostasis and ecosystem balance. Therefore, multidisciplinary approaches combining molecular biology, physiology, ecology, and computational modeling will be essential to translate HSF research into real-world impact.

In summary, HSFs are not merely heat-responsive transcription factors but dynamic integrators of environmental and developmental information. They represent a paradigm for how plants orchestrate complex networks to survive in challenging environments. As we continue to decode the systems biology of HSFs, we are also learning fundamental lessons about resilience, adaptability, and life’s capacity to thrive under stress. Harnessing these insights for agriculture will not only enhance food security but also deepen our understanding of biological robustness in the face of a rapidly changing planet.

## Figures and Tables

**Table 1:** Representative HSFs and Their Known Functions Across Plant Species.

Species	HSF Gene	Stress Type	References
*Arabidopsis thaliana*	*AtHSFA1a*	Heat	[96,114,115,116,117]
	*AtHSFA1b*	Heat, Drought, Salinity	
	*AtHSFA2*	Heat, Salinity	
	*AtHSFA7a*	Heat, Salinity	
	*AtHSFA8*	Oxidative stress	
	*AtHSFB1*	Heat, Salinity	
	*AtHSFC1*	Salinity	
*Solanum lycopersicum*	*SlHSFA1*	Heat	[86,118,119]
	*SlHSFA1a*	Drought	
	*SlHSFA2*	Heat	
	*SlHSFA3*	Salinity	
	*SlHSFA7*	Pathogen	
	*SlHSFB4a*	Pathogen	
*Zea mays*	*ZmHSF08*	Drought, Salinity	[98,104,120]
	*ZmHSF20*	Heat	
	*ZmHSF21*	Cold	
*Oryza sativa*	*OsHSFA3*	Cold	[88,92,105,121]
	*OsHSFA4d*	Cold	
	*OsHSFA9*	Cold	
	*OsHSFA7*	Salinity	
	*OsHSFB4a*	Cold	
	*OsHSFB4b*	Cold	
	*OsHSFB2b*	Salinity, drought	
	*OsHSFB4d*	Pathogen	
	*OsHSFC1*	Cold	
*Triticum aestivum*	*TaHSFA2d*	Salinity	[94,122]
	*TaHSFC2a*	Heat, Drought	

## Data Availability

No new data were created or analyzed in this study.
